# Supplementary material for: Impact of HPV mRNA types 16, 18, 45 detection on the risk of CIN3+ in young women with normal cervical cytology
Source: PLoS One. 2022 Nov 22;17(11):e0275858. doi: 10.1371/journal.pone.0275858 (PMC9681087; doi:10.1371/journal.pone.0275858)
Supplement: S1 Fig — (PDF) [file pone.0275858.s001.pdf]

Figure S1. Cumulative incidence ratio (CIR) of CIN3+ in the four groups for 5-7 years follow-up with confidence intervals (95% CI)

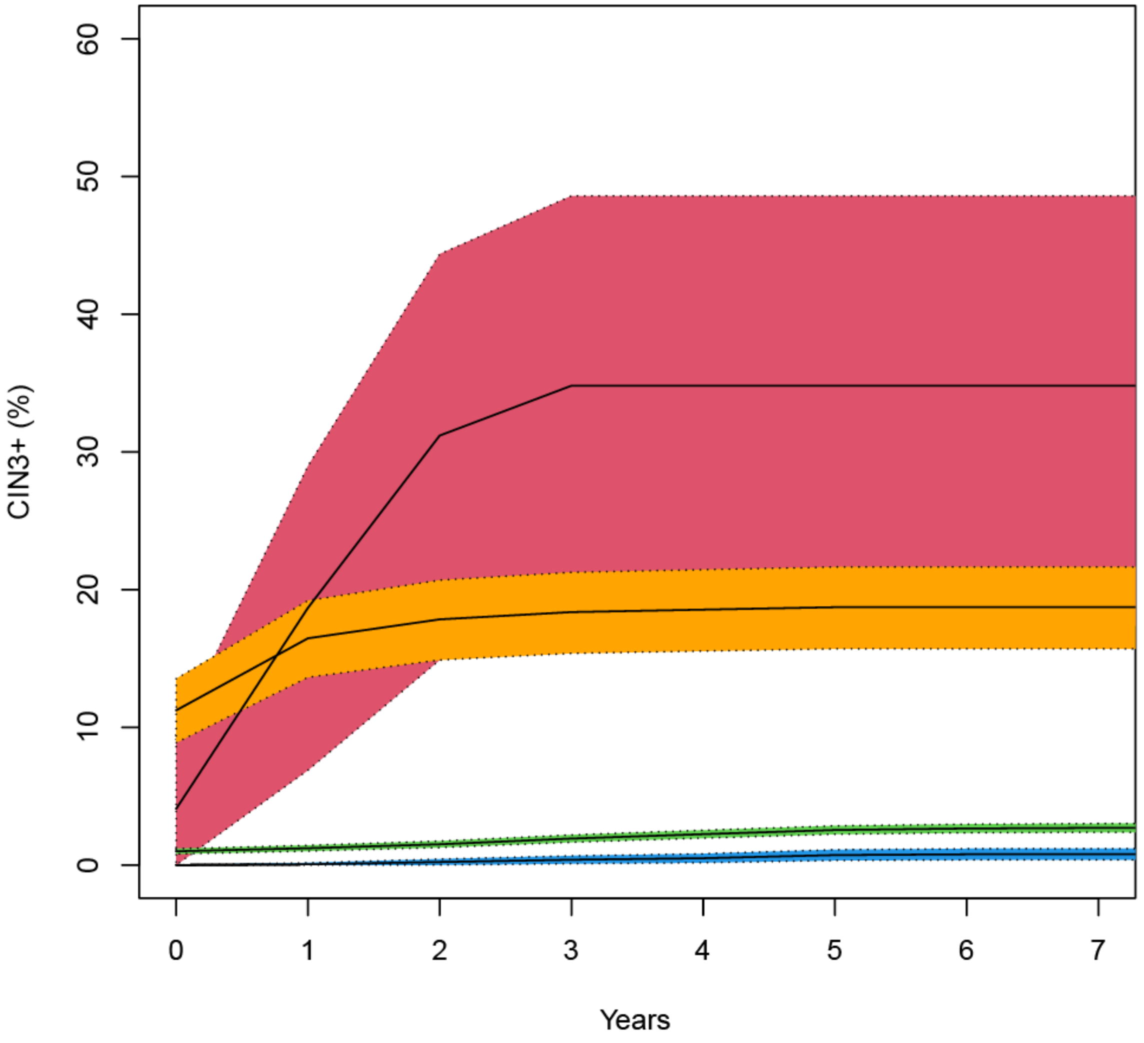

Red (Normal cytology / HPV mRNA positive): 34.8% (95% CI 17.3-48.6)

Orange (ASC-US+ / Not HPV-tested): 18.7% (95% CI 15.7 – 21.6)

Green (Normal cytology / Not HPV-tested): 2.7% (95% CI 2.4 – 3.0)

Blue (Normal cytology / HPV mRNA negative): 0.8% (95% CI 0.4-1.2)
