# Supplementary material for: Impact of HPV mRNA types 16, 18, 45 detection on the risk of CIN3+ in young women with normal cervical cytology
Source: PLoS One. 2022 Nov 22;17(11):e0275858. doi: 10.1371/journal.pone.0275858 (PMC9681087; doi:10.1371/journal.pone.0275858)
Supplement: S1 Table — (PDF) [file pone.0275858.s002.pdf]

Table S1 Category and risk of CIN3+ during follow-up (2014-2021)

Category	Number	CIN3+[1]	Risk of CIN3+ (%)	95% CI
Normal cytology, HPV mRNA negative	1 847	14	0.8[2]	0.4 – 1.2
Normal cytology, HPV mRNA positive	49	14	28.6	15.9 – 41.2
Normal cytology not HPV-tested	10 413	275	2.6	2.3 – 2.9
ASC-US+[3] not HPV-tested	712	126	17.7	14.9 – 20.5
Total	13 021	429	3.3	3.0 – 3.6

1) CIN3+ = cervical intraepithelial neoplasia grade 3 and worse (CIN3, ACIS and cancer)

2) Pearson Chi-Square 613.04, $P<0.001$

3) ASC-US+ = abnormal cytology (ASC-US, LSIL, AGUS, ASC-H, HSIL, ACIS and cancer)
