# Supplementary material for: Impact of HPV mRNA types 16, 18, 45 detection on the risk of CIN3+ in young women with normal cervical cytology
Source: PLoS One. 2022 Nov 22;17(11):e0275858. doi: 10.1371/journal.pone.0275858 (PMC9681087; doi:10.1371/journal.pone.0275858)
Supplement: S2 Table — (PDF) [file pone.0275858.s003.pdf]

Table S2 Characteristics of cancer cases

Baseline		Follow-up		
**Age**	**Cytology**	**Months to diagnosis**	**Histology**	**HPV DNA**
26	HSIL[1]	0	SCC[2]	NT[3]
28	LSIL[4]	35	SCC	16
29	HSIL	1	SCC	16
32	SCC	1	SCC	NT
33	HSIL	1	SCC	NT
37	HSIL	9	SCC	NT
26	Normal	0	ADC[5]	NT
28	Normal	38	SCC	16
30	Normal	41	ADC	16
30	Normal	35	ADC	18
31	Normal	53	ADC	NT
33	Normal	41	SCC	NT
39	Normal	73	ADC	16

1) HSIL = high-grade squamous intraepithelial lesion

2) SCC = squamous cell carcinoma

3) NT = not HPV tested

4) LSIL = low-grade squamous intraepithelial lesion

5) ADC = adenocarcinoma
