# Supplementary material for: Impact of HPV mRNA types 16, 18, 45 detection on the risk of CIN3+ in young women with normal cervical cytology
Source: PLoS One. 2022 Nov 22;17(11):e0275858. doi: 10.1371/journal.pone.0275858 (PMC9681087; doi:10.1371/journal.pone.0275858)
Supplement: Attachment — Submitted filename: 2022_apr_response_to_reviewers.docx [file pone.0275858.s005.docx]

**Response to reviewers**

***Academic Editor:*** *Please address the publication Criteria 3 by making sure study population and procedures are clearly described and unambiguous. (possible DNA testing overlap, biopsy results present while not indicated as necessary by repeat cytology.)*

**Our reply:** In the study period 2014-2017 there were no HPV DNA test in primary screening in our region of Norway. According to national guidelines there were no HPV DNA testing in women 25-39 years of age with normal cytology. From 1. January 2019 there have been a gradually introduction of HPV DNA test in primary screening of women 34-69 years.

All the women were followed-up according to national guidelines up to December 2021. All biopsies were included regardless of indication by cytology, HPV-test or clinical indication.

***Academic Editor:*** *Another critical issue is with Criteria 4, ie. point 4 raised by reviewer 1. Different results found in 0.8% or repeat testing in 2% cases doesn't suggest cost effectiveness of having to test 2300 samples by molecular methods. Possibly, additional data needs to be presented to justify the current conclusions.*

**Our reply:** It has been estimated that the Norwegian cervical cancer screening program each year cost 100 million USD, detecting 7.000 cases of CIN3, giving av cost of 14,300 USA per CIN3 detected. In our material 14 cases of CIN3 were detected among 1896 women with normal cytology tested by a 3-type HPV mRNA test which cost 15 USD per test, giving a cost of 2,030 USD per extra CIN3 detected, an approach considered as cost effective.

***Academic Editor:*** The introduction well describes that cytology is the primary screening tool in the population 25-33 years old. There were 39% cancer cases were identified in the population 25-39 years. Why did the authors decide to include women below 40? It might be more clear to also say “young women up to 39 years of age” (P4 L71) to avoid confusion, but more importantly, there were at least some women 34-39 for whom cytology was not the primary screening test as implied by the introduction? The population should be explicitly limited to women 25-39 that were only screened by cytology.

**Our reply:** The study includes women 25-39 years of age. The title, introduction, material, and methods are now reformulated to make this clearer.

***Academic Editor:*** *Upon initial re-screening based on mRNA testing, 9 cases were found to be high grade diagnosis (HSIL, ASC-H or AGUS) and 17 with low grade lesions. However, it is not clear why 31 biopsies were finally collected from 50 initial HPV positive cases. Were additional biopsies taken from any of the remaining 2332 women during the follow-up as well? Were some cases missed by mRNA testing as well based on the final outcomes?*

**Our reply:** Of the 13,021 women included 2014-2017, 429 women (3.3%) had CIN3+ confirmed by biopsy in the follow-up through December 2021, including 13 cases of invasive cervical cancer. Of the 1,896 women with normal cytology and HPV mRNA test (intervention group), 49 women (2.6%) had a positive test. The risk of CIN3+ of women with positive and negative HPV mRNA test were 28.6% (14/49) and 0.8% (14/1847). None of the women with HPV mRNA test developed cervical cancer in the follow-up. Of the 11,125 women with cytology only (control group), 712 women (6.4%) had abnormal cytology (ASC-US+). The risk of CIN3+ of women with abnormal and normal cytology were 17.7% (126/712) and 2.6% (275/10,413).

***Academic Editor:*** *P7 L149-154 These paragraphs appear to be figure legends. The information is inconsistent in presentation of otherwise similar figures. If initial cytology year is not shown, specifying followup year is not informative. For Fig 2a (left?) the legend implies this is index cytology image?*

**Our reply:** The paragraphs are now deleted.

***Reviewer #1:*** *By now, the use of HPV E6/E7 mRNA is consistently regarded as experimental, and no data are supporting its introduction in a screening setting*

**Our reply:** There are several publications supporting the use of HPV E6/E7 mRNA testing. Most publications regarding HPV E6/E7 mRNA testing are the use of a 5-type HPV mRNA test in triage of women with minor cervical lesions (ASC-US / LSIL), for example:

1.	Derbie A, Mekonnen D, Woldeamanuel Y, Van Ostade X, Abebe T. HPV E6/E7 mRNA test for the detection of high grade cervical intraepithelial neoplasia (CIN2+): a systematic review. Infect Agent Cancer. 2020 Feb 7;15:9. doi: 10.1186/s13027-020-0278-x. PMID: 32047531; PMCID: PMC7006188.

<https://pubmed.ncbi.nlm.nih.gov/32047531/>

2.	Sørbye SW, Fismen S, Gutteberg T, Mortensen ES. Triage of women with minor cervical lesions: data suggesting a "test and treat" approach for HPV E6/E7 mRNA testing. PLoS One. 2010 Sep 13;5(9):e12724. doi: 10.1371/journal.pone.0012724. PMID: 20856930; PMCID: PMC2938337.

<https://pubmed.ncbi.nlm.nih.gov/20856930/>

3.	Verdoodt F, Szarewski A, Halfon P, Cuschieri K, Arbyn M. Triage of women with minor abnormal cervical cytology: meta-analysis of the accuracy of an assay targeting messenger ribonucleic acid of 5 high-risk human papillomavirus types. Cancer Cytopathol. 2013 Dec;121(12):675-87. doi: 10.1002/cncy.21325. Epub 2013 Jul 23. PMID: 23881840.

<https://pubmed.ncbi.nlm.nih.gov/23881840/>

4.	Westre B, Giske A, Guttormsen H, Sørbye SW, Skjeldestad FE. 5-type HPV mRNA versus 14-type HPV DNA test: test performance, over-diagnosis and overtreatment in triage of women with minor cervical lesions. BMC Clin Pathol. 2016 Jun 7;16:9. doi: 10.1186/s12907-016-0032-x. PMID: 27279798; PMCID: PMC4897822.

<https://pubmed.ncbi.nlm.nih.gov/27279798/>

5.	Sørbye SW, Fismen S, Gutteberg TJ, Mortensen ES, Skjeldestad FE. HPV mRNA is more specific than HPV DNA in triage of women with minor cervical lesions. PLoS One. 2014 Nov 18;9(11):e112934. doi: 10.1371/journal.pone.0112934. PMID: 25405981; PMCID: PMC4236101.

<https://pubmed.ncbi.nlm.nih.gov/25405981/>

But there is also a publication using a 5-type HPV mRNA test in primary screening:

Sørbye SW, Fismen S, Gutteberg TJ, Mortensen ES, Skjeldestad FE. Primary cervical cancer screening with an HPV mRNA test: a prospective cohort study. BMJ Open. 2016 Aug 11;6(8):e011981. doi: 10.1136/bmjopen-2016-011981. PMID: 27515759; PMCID: PMC4985920.

https://pubmed.ncbi.nlm.nih.gov/27515759/

There is also a publication using the same 3-type HPV mRNA test in women with normal cytology:

Westre B, Giske A, Guttormsen H, Wergeland Sørbye S, Skjeldestad FE. Quality control of cervical cytology using a 3-type HPV mRNA test increases screening program sensitivity of cervical intraepithelial neoplasia grade 2+ in young Norwegian women-A cohort study. PLoS One. 2019 Nov 5;14(11):e0221546. doi: 10.1371/journal.pone.0221546. PMID: 31689301; PMCID: PMC6830931.

<https://pubmed.ncbi.nlm.nih.gov/31689301/>

***Reviewer #1:*** *it at least questionable to promote the routine use of mRNA detection in a screening setting were HPV-DNA detection is still to be introduced and analyzed.*

**Our reply:** HPV DNA testing is introduced to screening in several countries, but mostly in women 34-69 years of age (or women 30-65 years of age). Women 25-33 years are still recommended cervical cytology.

***Reviewer #1:*** *With the use of HPV-DNA testing as primary screening tool, the targeted population of the study (25-39 yrs.) would be significantly reduced to 25-30 yrs. women (only cytologically screened) and the relative importance of mRNA will undoubtfully decrease.*

**Our reply:** Due to the high positivity rate using a 14-type HPV DNA test in women 30-33 years of age, a combination of cytology and a 3-type HPV mRNA test will perform better in women 25-33 years of age.

***Reviewer #1:*** *The overall prevalence of mRNA HPV positive CIN2+ lesions reported in cytology-negative cases accounts for 0,8% (19 out of 2382 tested cases); this result seems to more support the good performance of cytology rather than a strong need for introducing a new tool.*

**Our reply:** Of the 13,021 women, 429 women (3.3%) had CIN3+ confirmed by biopsy in the follow-up, including 13 cases of invasive cervical cancer. Of the 429 women with CIN3+, only 126 had abnormal cytology at baseline. Of the 13 cases of cancer, only 7 had abnormal cytology at baseline. We do not think this shows good performance of cytology.

Of the 11,125 women with cytology only (control group), 712 women (6.4%) had abnormal cytology (ASC-US+). The risk of CIN3+ of women with abnormal and normal cytology were 17.7% (126/712) and 2.6% (275/10,413).

Of the 1,896 women with normal cytology and HPV mRNA test (intervention group), 49 women (2.6%) had a positive test. The risk of CIN3+ of women with positive and negative HPV mRNA test were 28.6% (14/49) and 0.8% (14/1847). None of the women with HPV mRNA test developed cervical cancer in the follow-up.

***Reviewer #1:*** *The authors underline that retesting cytology-negative cases of the specified age groups does not represent an excessive workload; when both the procedures are together considered, assuming that around 2% of all screened cases will undergo mRNA HPV + repeated cytology, this statement appears to be poorly acceptable.*

**Our reply:** Using a 14-type HPV DNA test in women 25-29 years of age, about 30% will have a positive test. It is not possible to do a targeted rescreening when 30% of all smears have to be rescreened. Using a 3-type HPV mRNA test, only 2.6% of the women 25-39 years with normal cytology had a positive HPV mRNA test. Rescreening 2.6% of the smears generates less workload than rescreening 30% of the smears.

***Reviewer #2:*** *The study consists in analyzing women with normal cytology and HPV mRNA assay positive. They found 50 women with these characteristics and their cytology was re-examined. Twenty-six had a new diagnosis: 11 ASC US, 6 LSIL, 6 ASC-H, 2HSIL and 1 AGUS. So only 9 had high risk cytology and should be managed with colposcopy and biopsy. The Results describe 31 histology analyses. Why? Why was performed biopsy for the other 22 women with low risk cytology result?*

**Our reply:** According to the Norwegian national guidelines, women with minor cervical lesions (ASC-US / LSIL) should be triaged by an HPV DNA test. Most of the women with a positive HPV mRNA test will also have a positive HPV DNA test.

**Reviewer #2:** The Authors say that women had <40 years, but they should be stratified according to their age. The clinical importance of earlier identification of these lesions may be limited because most CIN2 lesions, mostly in younger women, regress in a spontaneous way. Also women with CIN3, different studies indicate that about 30% of these lesion will assume a neoplastic phenotype in about 30 years.

**Our reply:** We use histologically confirmed CIN3+ as study endpoint. The PPV for CIN3+ was higher for women with normal cytology and a positive HPV mRNA test (28.6%) than women with abnormal cytology (17.7%). Women 25-39 years of age with CIN3 caused by HPV type 16, 18 or 45 should be treated. In our study there were 13 cases of cervical cancers in the follow-up. Eleven out of the 13 women with cancer were 25-33 years old at baseline. Seven out of the 13 women had normal cytology at baseline. None of the women tested by the 3-type HPV mRNA test developed cervical cancer in the follow-up.

**Fra:** em.pone.0.78eb22.ca0aa06b@editorialmanager.com <em.pone.0.78eb22.ca0aa06b@editorialmanager.com> **På vegne av** PLOS ONE
**Sendt:** tirsdag 25. januar 2022 18:44
**Til:** Sørbye Sveinung Wergeland <Sveinung.Wergeland.Sorbye@unn.no>
**Emne:** PLOS ONE Decision: PONE-D-21-34573 - [EMID:7994d4415d51163c]

PONE-D-21-34573
3-type HPV mRNA test in detection of CIN2+ in young women with normal cervical cytology
PLOS ONE

Dear Dr. Sørbye,

Thank you for submitting your manuscript to PLOS ONE. After careful consideration, the current manuscript cannot be accepted at this time. However, the topic is interesting and we would consider a re-submission.

Specifically:

Please address the publication Criteria 3 by making sure study population and procedures are clearly described and unambiguous. (possible DNA testing overlap, biopsy results present while not indicated as necessary by repeat cytology.)

Another critical issue is with Criteria 4, ie. point 4 raised by reviewer 1. Different results found in 0.8% or repeat testing in 2% cases doesn't suggest cost effectiveness of having to test 2300 samples by molecular methods. Possibly, additional data needs to be presented to justify the current conclusions.

I am sorry that we cannot be more positive on this occasion, but hope that you appreciate the reasons for this decision.

Yours sincerely,

Ivan Sabol
Academic Editor
PLOS ONE

Additional Editor Comments:

The manuscript appears to be well written, however there is some confusion about the methods.
The introduction well describes that cytology is the primary screening tool in the population 25-33 years old. There were 39% cancer cases were identified in the population 25-39 years. Why did the authors decide to include women below 40? It might be more clear to also say “young women up to 39 years of age” (P4 L71) to avoid confusion, but more importantly, there were at least some women 34-39 for whom cytology was not the primary screening test as implied by the introduction? The population should be explicitly limited to women 25-39 that were only screened by cytology.
P4L87-89. Single sentence single paragraph section should be joined with the preceding section.

Upon initial re-screening based on mRNA testing, 9 cases were found to be high grade diagnosis (HSIL, ASC-H or AGUS) and 17 with low grade lesions. However, it is not clear why 31 biopsies were finally collected from 50 initial HPV positive cases. Were additional biopsies taken from any of the the remaining 2332 women during the follow-up as well? Were some cases missed by mRNA testing as well based on the final outcomes?
P7 L149-154 These paragraphs appear to be figure legends. The information is inconsistent in presentation of otherwise similar figures. If initial cytology year is not shown, specifying followup year is not informative. For Fig 2a (left?) the legend implies this is index cytology image?


[Note: HTML markup is below. Please do not edit.]

Reviewers' comments:

Reviewer's Responses to Questions

**Comments to the Author**

1. Is the manuscript technically sound, and do the data support the conclusions?

The manuscript must describe a technically sound piece of scientific research with data that supports the conclusions. Experiments must have been conducted rigorously, with appropriate controls, replication, and sample sizes. The conclusions must be drawn appropriately based on the data presented.

Reviewer #1: Partly

Reviewer #2: No

2. Has the statistical analysis been performed appropriately and rigorously?

Reviewer #1: Yes

Reviewer #2: N/A

3. Have the authors made all data underlying the findings in their manuscript fully available?

The [PLOS Data policy](http://www.plosone.org/static/policies.action#sharing) requires authors to make all data underlying the findings described in their manuscript fully available without restriction, with rare exception (please refer to the Data Availability Statement in the manuscript PDF file). The data should be provided as part of the manuscript or its supporting information, or deposited to a public repository. For example, in addition to summary statistics, the data points behind means, medians and variance measures should be available. If there are restrictions on publicly sharing data—e.g. participant privacy or use of data from a third party—those must be specified.

Reviewer #1: Yes

Reviewer #2: Yes

4. Is the manuscript presented in an intelligible fashion and written in standard English?

PLOS ONE does not copyedit accepted manuscripts, so the language in submitted articles must be clear, correct, and unambiguous. Any typographical or grammatical errors should be corrected at revision, so please note any specific errors here.

Reviewer #1: Yes

Reviewer #2: Yes

5. Review Comments to the Author

Please use the space provided to explain your answers to the questions above. You may also include additional comments for the author, including concerns about dual publication, research ethics, or publication ethics. (Please upload your review as an attachment if it exceeds 20,000 characters)

Reviewer #1: The manuscript focuses the interesting topic of detecting specific hrHPV mRNA in the preventive strategy for cervical cancer. it is worldwide accepted that hrHPV-DNA integration is the major causal factor for cancer development, and mRNA detection is the ultimate biomarker developed for the progression towards cancer. The authors studied the application of mRNA detection within the screening strategy and concluded that its application in cytologically-negative cases of <40 yrs. of age would represent a significant improvement in screening efficacy.
The manuscript appears to be well written and presented and results clearly detailed.
Nevertheless some flaws need to be addressed:
- By now, the use of HPV E6/E7 mRNA is consistently regarded as experimental, and no data are supporting its introduction in a screening setting
- it at least questionable to promote the routine use of mRNA detection in a screening setting were HPV-DNA detection is still to be introduced and analyzed.
- With the use of HPV-DNA testing as primary screening tool, the targeted population of the study (25-39 yrs.) would be significantly reduced to 25-30 yrs. women (only cytologically screened) and the relative importance of mRNA will undoubtfully decrease.
- The overall prevalence of mRNA HPV positive CIN2+ lesions reported in cytology-negative cases accounts for 0,8% (19 out of 2382 tested cases); this result seems to more support the good performance of cytology rather than a strong need for introducing a new tool.
- The authors underline that retesting cytology-negative cases of the specified age groups does not represent an excessive workload; when both the procedures are together considered, assuming that around 2% of all screened cases will undergo mRNA HPV + repeated cytology, this statement appears to be poorly acceptable.
Despite the interesting amount of data reported, the manuscript does not reach a quality level consistent with a publication suggestion.

Reviewer #2: The paper of Shibli et al. considers a topic already performed in part by the Authors.
Even if interesting, I have major comments which should be addressed by the Authors:
1. The study consists in analyzing women with normal cytology and HPV mRNA assay positive.
They found 50 women with these characteristics and their cytology was re-examined. Twenty-six had a new diagnosis: 11 ASC US, 6 LSIL, 6 ASC-H, 2HSIL and 1 AGUS. So only 9 had high risk cytology and should be managed with colposcopy and biopsy. The Results describe 31 histology analyses. Why? Why was performed biopsy for the other 22 women with low risk cytology result?
2. The Authors say that women had <40 years, but they should be stratified according to their age.
The clinical importance of earlier identification of these lesions may be limited because most CIN2 lesions, mostly in younger women, regress in a spontaneous way.
Also women with CIN3, different studies indicate that about 30% of these lesion will assume a neoplastic phenotype in about 30 years.

6. PLOS authors have the option to publish the peer review history of their article ([what does this mean?](https://journals.plos.org/plosone/s/editorial-and-peer-review-process#loc-peer-review-history)). If published, this will include your full peer review and any attached files.


If you choose “no”, your identity will remain anonymous but your review may still be made public.


**Do you want your identity to be public for this peer review?** For information about this choice, including consent withdrawal, please see our [Privacy Policy](https://www.plos.org/privacy-policy).

Reviewer #1: No

Reviewer #2: No

[NOTE: If reviewer comments were submitted as an attachment file, they will be attached to this email and accessible via the submission site. Please log into your account, locate the manuscript record, and check for the action link "View Attachments". If this link does not appear, there are no attachment files.]

- - - - -
For journal use only: PONEDEC3
