# Supplementary material for: Impact of HPV mRNA types 16, 18, 45 detection on the risk of CIN3+ in young women with normal cervical cytology
Source: PLoS One. 2022 Nov 22;17(11):e0275858. doi: 10.1371/journal.pone.0275858 (PMC9681087; doi:10.1371/journal.pone.0275858)
Supplement: Attachment — Submitted filename: 2022_08_Response to Reviewers_v2.docx [file pone.0275858.s006.docx]

Academic Editor: However, there are again some inconsistencies that are confusing for readers not familiar with the Norwegian screening program or where authors should more clearly present information. For example P4L83 states there were 10724 women with cytology only however P6L130 says there were 11125. Since The table 1 is more in line with P6L130 apparently there is some error in the text at P4

Our response: The correct number is 11,125. The numbers in the manuscript are now updated.

Academic Editor: P5 L106 only follow-up of women with positive mRNA test is described. It is uncertain how other women were followed up? When stating “Women with revised cytological diagnoses were followed-up in line with the Norwegian national guidelines until December 2021” introduces uncertainty since it is unknown how this revised cytological diagnosis was considered within this manuscript. The sentence should be revised to explain that all women were followed up and not only women with revised cytology.

Our response: The sentence is now revised. In the study period, the Norwegian Cervical Program recommends women aged 25-69 to be screened with cervical cytology every three years. Women with high-grade cytology are recommended colposcopy and biopsy. Women with low-grade cytology are triaged by an HPV test. Women with normal cytology and a positive HPV-test are recommended follow-up after 12 months.

Academic Editor: Out of 49 cases with mRNA positive result, how many cases had revised diagnosis and was it a reexamination of the initial slide or collection of a new slide for mRNA positive women? How many cases were true cytology negative (remain negative) and how many were false negative (revised to abnormal) after revision? While actual grades (revision from normal to some stage of abnormal) might be irrelevant, some summary of how many times cytology performed sub optimally (if it is a reexamination of the existing slide) would be useful.

Our response: The cytology diagnoses were revised in 53.1% (26/49) of the HPV mRNA positive cases. 23 cases were true cytology negative. The result section is now updated.

Academic Editor: Further information on how follow up is actually done is needed. For example P6L138 implies the 3 years gap between testing, but figure 3 implies most CIN3+ events happened within 3 years even for cytology normal cases.

Our response: We have now updated the manuscript. Women with normal cytology (true cytology negative) and a positive HPV-test are recommended follow-up with another HPV-test after 12 months. Women with repeated positive HPV-tests are recommended colposcopy and biopsy. Women with a negative biopsy (Normal / CIN1) are recommended new cytology and HPV-test after 12 months. Even though women with normal cytology are recommended screening every three years, some women take cytology test every year.

Academic Editor: The text at P6 and Table 1 misuses the term PPV which often stands for positive predictive value; PPV = 100xTrue Positive/(True Positive +False Positive)). It is not a simple percentage

Our response: We have now reformulated. The correct term is “risk of CIN3+” in the follow-up in different subgroups.

Academic Editor: Table 1 and Figures 1, 2 and 3 essentially show the same data (0.8%, 2.6%, 17.7% and 28.6%). While it is clear that Figure 2 aims to visually show the difference in risk at specific branching points of the study, it could benefit from a more significant legend guiding the readers through the figure.

Our response: We have now reformulated, added 95% CI, p-values and moved table 1 to supplemental.

Reviewer #1: This is an interesting paper from a study which examines whether using an mRNA HPV test following a cytology negative result in women <40 years screened as part of the Norwegian cervical cancer screening programme could increase screening sensitivity, identifying more women who are at risk of developing CIN3+.

Reviewer #1: However, not all Figures are needed or clearly presented and correctly labelled. The methods do not contain sufficient details about the screening programme and the follow-up of women in the study including the small group who receive a positive HPV result following normal cytology who have their sample re-examined. The strengths and limitations of the study are not discussed.

Our response: We have now updated the labels of the figures. We have included more details of the Norwegian cervical cancer screening program and follow-up. The discussion is updated with strengths and limitations.

Reviewer #1: I would suggest rewording the title. It doesn’t make sense grammatically or describe the study very clearly.

Our response: The title is now updated.

Reviewer #1: Line 44-46. Consider changing to ‘HPV testing is performed for the early detection of cervical disease, both as a reflex test following cytology or as a primary screening method.’

Our response: Done.

Reviewer #1: Line 47. Specificity for what? i.e. for identifying women with high-grade lesions, CIN, cancer or something else?

Our response: Specificity for identifying women with high-grade lesions and cancer (CIN3+). Now reformulated.

Reviewer #1: Line 52. You say ‘by 2022’ but it is already mid-way through. Do you mean the end of 2022? Was it previously cytology based (I assume?)

Our response: By the end of 2022 HPV DNA test will replace cervical cytology in screening of women 34-69 years of age in Norway. Actually, HPV DNA primary screening was implemented in large parts of Norway from 1. July 2021, after a pilot in some counties which started in 2016.

Reviewer #1: Line 55. I think you mean that it’s not just that the screening programme hasn’t transitioned to primary HPV testing for this group but that cytology will remain the primary screening tool for this younger age group?

Our response: Yes. Reformulated.

Reviewer #1: Line 58. It is normal practice to avoid starting a sentence with a number.

Our response: Reformulated.

Reviewer #1: Line 59. I believe that terminology is moving away from using ‘smear’.

Our response: Yes. We did not use conventional PAP-smear, but liquid based cytology (LBC).

Reviewer #1: Line 62-64. Consider rephrasing this last sentence as it is crucial here that the reader understands your main point: i.e., ‘younger women are screened using cytology – given the high number of precancerous lesions in this group an mRNA reflex test following a negative cytology result could be used as a quality assurance measure to increase screening sensitivity’.

Our response: Done.

Reviewer #1: Line 66: there should be no apostrophe in HPVs.

Our response: Corrected.

Reviewer #1: Line 70: you use the word ‘proven’. Suggest rewording to ‘shown’ or ‘found’

Our response: Done.

Reviewer #1: Line 71: consider using a different word than ‘tool’

Our response: Reformulated.

Reviewer #1: Line 72: ‘Elevated risk of future’. In line 62 you infer they are ‘high risk’ but here you state low-risk population.

Our response: It is about risk stratification. Women with normal cytology should be a low-risk group. However, cervical cytology is not perfect. We use the HPV mRNA test to identify the few women with high risk of CIN3+ among women with normal cytology.

Reviewer #1: Line 73: Consider rewording this for clarity. Also, avoid the use of ‘we’ but state as ‘the aim of this study was…’.

Our response: Reformulated.

Reviewer #1: I understand from the methods section that your baseline samples are taken as part of routine screening in 2014-2017. However, you do not state how long the interval is between routine screens for women of this age or what happens to this interval if they have a positive cytology result.

Our response: We have now updated the manuscript. In the study period, the Norwegian Cervical Program recommends women aged 25-69 to be screened with cervical cytology every three years. Women with high-grade cytology are recommended colposcopy and biopsy. Women with low-grade cytology are triaged by an HPV-test. Women with normal cytology and a positive HPV-test are recommended follow-up after 12 months. Even though women with normal cytology (and no HPV-test) are recommended screening every three years, some women take cytology test every year.

Reviewer #1: Line 108: Your main outcome measure is CIN3+. But I do not understand from the Methods how this outcome is measured, when and in whom? Everyone presumably? But as part of their subsequent screen – and if that’s the case, surely not everyone has a subsequent screen? Some must be lost to follow up? In the Results you mention ‘follow-up through 2021’ but you need to explain more in the Methods what the follow-up means.

Our response: Our laboratory is the only laboratory in Nordland county. We receive screening samples from all women who attend screening in our county. The Norwegian Cervical Program writes personal invitations to all women in screening age. If the women do not attend screening, they get a reminder. The coverage of the screening program is 70% after three years and 80% after five years. In addition, our lab has contacted the physician to ensure follow-up of abnormal screening results. Of women with index cytology in 2014-2017, almost 100% have a subsequent screen of follow-up within the end of 2021.

Reviewer #1: Line 106: I think you are saying that in women with a positive mRNA HPV result their index cytology (originally deemed as ‘normal’) is re-examined by a technician. But you do not then present the numbers of how many are deemed as ‘normal’ when re-examined? What then happens to those with an ‘abnormal’ result on re-examination in terms of their screening internal? Colposcopy? What is their CIN3+ outcome? And what happens or would happen to those who are HPV +ve but cytology ‘normal’ on second reading, is the interval to their next screen shorter?

Our response: We have now updated the manuscript. The cytology diagnoses were revised from normal to abnormal in 53.1% (26/49) of the HPV mRNA positive cases, while 23 cases remained normal after revision (true cytology negative). Women with abnormal cytology and a positive HPV-test are referred to colposkopi and biopsy. Women with normal cytology (despite re-examination) and a positive HPV mRNA test are followed-up with a new cytology and HPV-test after 12 months. Women with repeated positive HPV-test (HPV pos x 2) are referred to colposcopy and biopsy.

Reviewer #1: Figure 1: You split by the group that have mRNA testing and the group that don’t. Have you considered splitting first by cytology normal and cytology abnormal, then splitting the cytology normal into those tested for HPV and those not – this makes a more intuitive tree – but ends up with the same four groups represented at the bottom? The diagram needs and title and footnotes to explain abbreviations.

Our response: We have tried different ways of splitting, but after a few rounds of trial and error, we have chosen to emphasise the intervention group (Cytology normal & HPV mRNA tested) and control group (Cytology only – not HPV mRNA tested). The flowchart is now updated with new labels and footnotes.

Reviewer #1: Line 136: Correction ‘a short time’

Our response: Done.

Reviewer #1: Line 138: Correction ‘nest’ to ‘next’

Our response: Done.

Reviewer #1: Figure 2: I have no idea what this figure means. It doesn’t add anything to the results so consider removing.

Our response: Philip Castle et al. introduced the concept of risk-based screening in 2007. Women with equal risk of CIN3+ should be managed equally. Our Figure 2 is based on Figure 1 “Risk of cervical precancer and results of screening and clinical management for cervical cancer prevention” in the publication Castle et al. 2007.

Castle PE, Sideri M, Jeronimo J, et al. Risk assessment to guide the prevention of cervical cancer. Am J Obstet Gynecol 2007;197:356.e1-356.e6.

<https://pubmed.ncbi.nlm.nih.gov/17904958/>

We have used a similar risk diagram in another publication (Sørbye et al. 2011), and we think it is very illustrative to show the difference in risk of CIN3+ at specific branching points of the study. However, we have updated the legends for clarity.

Sørbye SW, Arbyn M, Fismen S, Gutteberg TJ, Mortensen ES. HPV E6/E7 mRNA testing is more specific than cytology in post-colposcopy follow-up of women with negative cervical biopsy. PLoS One. 2011;6(10):e26022. doi: 10.1371/journal.pone.0026022. Epub 2011 Oct 6. PMID: 21998748; PMCID: PMC3188582.

<https://pubmed.ncbi.nlm.nih.gov/21998748/>

Reviewer #1: Figure 3: Without knowing anything about the follow-up of these women, the screening cycles etc. this seems hard to interpret. The labeling seems insufficient, for example these four groups are actually 1) normal cytology + mRNA HPV positive 2) abnormal cytology (no HPV testing) 3) Normal cytology (no HPV testing) 4) normal cytology + mRNA HPV negative. There is no info in the methods about how these data were obtained or what the graph represents so consider removing this or labeling better.

Our response: Women with normal cytology (and no HPV-test) are recommended screening every three years. Women with high-grade cytology (primary or after re-examination) are recommended colposcopy and biopsy. Women with low-grade cytology are triage by HPV-test. Women with normal cytology and a positive HPV-test are recommended follow-up with a new cytology and HPV-test after 12 months. Women with repeated positive HPV-test (HPV x 2), are recommended colposcopy and biopsy. Women with a negative biopsy (Normal / CIN1) are recommended follow-up with a new cytology and HPV-test after 12 months. It is correct that the four groups are as reviewer 1 state. We have updated the labeling.

Reviewer #1: Table 1: See my comment about labeling of Figure 3. You do not mention this table in the Results. I do not think that the label PPV is correct. PPV in a clinical setting means what is the probability, given your positive result, that you really are positive. The cytology ‘abnormal’ result would be considered a ‘positive’ result as would the mRNA HPV positive group, but the other groups would be considered as a ‘negative’ result. I think what these values really are is the percentage who go on to develop CIN3+ within 7 years of the baseline result (i.e. 2014-2021).

Our response: Yes. You are right. We have moved table 1 to supplemental and added 95% CI and p-values.

Reviewer #1: Line 173: why is this in quotations?

Our response: The text was in quotations because it is a quote. We have now reformulated with our own wordings.

Reviewer #1: Line 177: consider using ‘intense’ rather than aggressive?

Our response: Done.

Reviewer #1: You do not discuss the strengths or limitations of the study. For example, the fact that there is no statistical analysis.

Our response: We have added some statistical analysis. We have also included strengths and limitations of the study.

Reviewer #1: Using mRNA HPV testing as a reflex test is only one way to change the screening pathway for this age group, it might be appropriate to suggest alternatives (such as AI to improve cytology) or suggest future research e.g. a cost-effectiveness analysis.

Our response: Yes. AI may improve cytology. However, of the 14 women with CIN3+ found by HPV mRNA test, only 8 cases were among the 26 revised diagnoses (abnormal cytology after re-examination), and while 6 cases were among the 23 cases which remained normal (“true cytology negative”). In an unpublished quality study from Belgium using the same 3-type HPV mRNA test in women with normal cytology using computer-aided screening, the HPV mRNA positivity rate was the same as in our study, indication that computer-aided screening not necessary will improve the quality significantly.

Reviewer #1: I’m not clear on what you are suggesting happens to women who are found cytology normal mRNA HPV positive – colposcopy or a shorter screening interval?

Our response: According to Norwegian national guidelines, women with normal cytology and a positive HPV-test are recommended follow-up with a new cytology and HPV-test after 12 months. Women with repeated positive HPV-test (HPV pos x 2) are recommended colposcopy and biopsy. The manuscript is now updated.

Reviewer #1: Line 210: You say 2.6% of cases in our study needed to be rescreened – i.e. 2.6% of samples with normal cytology but positive mRNA HPV have their original sample re-evaluated by a technician. Using the word ‘rescreened’ suggests they provide a new sample, but I don’t think from the methods that this is the case.

Our response: Yes. The same index cytology was re-evaluated by a different technician than the first technician who evaluated the index cytology. This is now described in the manuscript.

Reviewer #1: Line 218: There is no excessive workload in terms of the number of samples that need to be re-examined but if HPV testing were used as a reflex test then every cytology normal sample would need a reflex HPV test and this would increase the workload in the labs presumably as well as the cost of screening.

Our response: Yes. Co-testing (cytology + HPV-testing) requires more resources than cytology alone, but HPV-testing is usually automatized with little “hands-on-time”. The most time-consuming is manual evaluation of cytology slides in microscope (both primary and re-evaluation). Most of the normal cytology slides are evaluated by a single technician, but some laboratories have rapid pre-screening or rapid re-screening to try to reduce the risk of abnormal cells being misinterpreted or overlooked. During the study period, our lab started double screening of all cytology samples using two independent technicians for each cytology sample, but this neither reduced the HPV mRNA positivity rate in women with normal cytology, nor reduced the risk of CIN3+ in the follow-up of women with a positive HPV mRNA test.

Reviewer #2: This study showed that three type HPV mRNA test increases the sensitivity in cervical cancer screening program without an excessive workload. This is interesting and important study to prevent cervical cancer incidence in young women. The authors also said that detection of three HPV types (HPV types 16, 18, and 45) in this assay might be best choice in the screening of women of 25-39 years of age, since some previous studies suggested that 90% of cervical cancer are positive with HPV16, 18 or 45 in this age group.

Our response: There is always a trade-off for sensitivity and specificity and different positivity rate of the screening test. Using a 14-type HPV DNA test will give too many positive samples, and rescreening will not be as targeted as using av 3-type HPV mRNA test. Using a mRNA test for HPV type 16, 18 and 45 will not find CIN3+ caused by other HPV-types in women with normal cytology, but the risk of cervical cancer is lower for other HPV-types, especially in young women.

Reviewer #2: However, this reviewer does not convince with this hypothesis that this three-HPV mRNA test is the best choice for the above mentioned purpose, since this study showed that the sensitivity of CIN3+ in the intervention group was merely 50% (14/28), although the sensitivity was high enough as it was 97.4% (1847/1896). The authors are likely to say no cancer were found in these CIN3+ cases in the intervention group. However, to prove this assay is the best choice, the authors should show most of 13 cervical cancer cases in the control group in this study are positive by this assay. If possible, they also should examine what HPV types are positive in 14 CIN3+ cases showing negative with this HPV test in the intervention group. Such data may be important to support the findings.

Our response: We do not have the residual material of the index cytology of samples from 2014-2017. Of the 13 women with cervical cancer (not HPV mRNA tested at baseline) there were five HPV type 16, one HPV type 18, and seven not HPV-tested in the follow-up including two women who died of cervical cancer.

Of the 14 CIN3+ with a negative 3-type HPV mRNA test at baseline, there were two HPV 16, two HPV 18, five HPV “others” (not HPV 16/18), two HPV pos (not specified) and three women were not HPV-tested before the CIN3+ diagnosis. For seven of the 14 women, the CIN3+ was detected 4-6 years after index cytology and can be a result of a new HPV-infection after baseline.

**Fra:** em.pone.0.7d3a17.90c5987e@editorialmanager.com <em.pone.0.7d3a17.90c5987e@editorialmanager.com> **På vegne av** PLOS ONE
**Sendt:** tirsdag 9. august 2022 20:41
**Til:** Sørbye Sveinung Wergeland <Sveinung.Wergeland.Sorbye@unn.no>
**Emne:** PLOS ONE Decision: Revision required [PONE-D-22-12278] - [EMID:4dedda8d86037608]

PONE-D-22-12278

3-type HPV mRNA test in detection of CIN3+ in women 25-39 years with normal cervical cytology

PLOS ONE

Dear Dr. Sørbye,

Thank you for submitting your manuscript to PLOS ONE. After careful consideration, we feel that it has merit but does not fully meet PLOS ONE’s publication criteria as it currently stands. Therefore, we invite you to submit a revised version of the manuscript that addresses the points raised during the review process.

The resubmitted manuscript appears more streamlined than before, however, readers unfamiliar with Norwegian screening program would struggle to understand some aspects of the work described.

There are also some data discrepancies in the text that might be typos but could be confusing. Furthermore please aim to reduce the overlap of figures and tables shown. Essentially the same data is given 4 times plus the text. Personally table 1 seems most superfluous since Figure 1 contains info on the full population, figure 2 is quite illustrative (but needs a thorough legend) and figure 3 provides additional time data. Unless mRNA test can be done subsequently on the invasive cancer cases shown in Table 2 as suggested by Reviewer 2, this table could also be moved to the supplement material

Please submit your revised manuscript by Sep 22 2022 11:59PM. If you will need more time than this to complete your revisions, please reply to this message or contact the journal office at plosone@plos.org. When you're ready to submit your revision, log on to <https://www.editorialmanager.com/pone/> and select the 'Submissions Needing Revision' folder to locate your manuscript file.

Please include the following items when submitting your revised manuscript:

- A rebuttal lettedr that responds to each point raised by the academic editor and reviewer(s). You should upload this letter as a separate file labeled 'Response to Reviewers'.
- A marked-up copy of your manuscript that highlights changes made to the original version. You should upload this as a separate file labeled 'Revised Manuscript with Track Changes'.
- An unmarked version of your revised paper without tracked changes. You should upload this as a separate file labeled 'Manuscript'.

If you would like to make changes to your financial disclosure, please include your updated statement in your cover letter. Guidelines for resubmitting your figure files are available below the reviewer comments at the end of this letter.

If applicable, we recommend that you deposit your laboratory protocols in protocols.io to enhance the reproducibility of your results. Protocols.io assigns your protocol its own identifier (DOI) so that it can be cited independently in the future. For instructions see: <https://journals.plos.org/plosone/s/submission-guidelines#loc-laboratory-protocols>. Additionally, PLOS ONE offers an option for publishing peer-reviewed Lab Protocol articles, which describe protocols hosted on protocols.io. Read more information on sharing protocols at <https://plos.org/protocols?utm_medium=editorial-email&utm_source=authorletters&utm_campaign=protocols>.

We look forward to receiving your revised manuscript.

Kind regards,

Ivan Sabol
Academic Editor
PLOS ONE

Journal Requirements:

When submitting your revision, we need you to address these additional requirements.

1. Please ensure that your manuscript meets PLOS ONE's style requirements, including those for file naming. The PLOS ONE style templates can be found at 
<https://journals.plos.org/plosone/s/file?id=wjVg/PLOSOne_formatting_sample_main_body.pdf> and 
<https://journals.plos.org/plosone/s/file?id=ba62/PLOSOne_formatting_sample_title_authors_affiliations.pdf>

Additional Editor Comments:

The resubmitted manuscript appears to be much more streamlined version that now starts with a great numbed of cases and further elaborates a subset in more detail. This gives the readers a much broader view of the situation and presents a valuable dataset.
However, there are again some inconsistencies that are confusing for readers not familiar with the Norwegian screening program or where authors should more clearly present information.

For example P4L83 states there were 10724 women with cytology only however P6L130 says there were 11125. Since The table 1 is more in line with P6L130 apparently there is some error in the text at P4
P5 L106 only follow-up of women with positive mRNA test is described. It is uncertain how other women were followed up? When stating “Women with revised cytological diagnoses were followed-up in line with the Norwegian national guidelines until December 2021” introduces uncertainty since it is unknown how this revised cytological diagnosis was considered within this manuscript. The sentence should be revised to explain that all women were followed up and not only women with revised cytology.
Out of 49 cases with mRNA positive result, how many cases had revised diagnosis and was it a reexamination of the initial slide or collection of a new slide for mRNA positive women? How many cases were true cytology negative (remain negative) and how many were false negative (revised to abnormal) after revision? While actual grades (revision from normal to some stage of abnormal) might be irrelevant, some summary of how many times cytology performed sub optimally (if it is a reexamination of the existing slide) would be useful.

Further information on how follow up is actually done is needed. For example P6L138 implies the 3 years gap between testing, but figure 3 implies most CIN3+ events happened within 3 years even for cytology normal cases.
The text at P6 and Table 1 misuses the term PPV which often stands for positive predictive value; PPV = 100xTrue Positive/(True Positive +False Positive)). It is not a simple percentage
Table 1 and Figures 1, 2 and 3 essentially show the same data (0.8%, 2.6%, 17.7% and 28.6%). While it is clear that Figure 2 aims to visually show the difference in risk at specific branching points of the study, it could benefit from a more significant legend guiding the readers through the figure.

[Note: HTML markup is below. Please do not edit.]

Reviewers' comments:

Reviewer's Responses to Questions

**Comments to the Author**

1. Is the manuscript technically sound, and do the data support the conclusions?

The manuscript must describe a technically sound piece of scientific research with data that supports the conclusions. Experiments must have been conducted rigorously, with appropriate controls, replication, and sample sizes. The conclusions must be drawn appropriately based on the data presented.

Reviewer #1: Yes

Reviewer #2: Yes

2. Has the statistical analysis been performed appropriately and rigorously?

Reviewer #1: N/A

Reviewer #2: Yes

3. Have the authors made all data underlying the findings in their manuscript fully available?

The [PLOS Data policy](http://www.plosone.org/static/policies.action#sharing) requires authors to make all data underlying the findings described in their manuscript fully available without restriction, with rare exception (please refer to the Data Availability Statement in the manuscript PDF file). The data should be provided as part of the manuscript or its supporting information, or deposited to a public repository. For example, in addition to summary statistics, the data points behind means, medians and variance measures should be available. If there are restrictions on publicly sharing data—e.g. participant privacy or use of data from a third party—those must be specified.

Reviewer #1: Yes

Reviewer #2: Yes

4. Is the manuscript presented in an intelligible fashion and written in standard English?

PLOS ONE does not copyedit accepted manuscripts, so the language in submitted articles must be clear, correct, and unambiguous. Any typographical or grammatical errors should be corrected at revision, so please note any specific errors here.

Reviewer #1: Yes

Reviewer #2: Yes

5. Review Comments to the Author

Please use the space provided to explain your answers to the questions above. You may also include additional comments for the author, including concerns about dual publication, research ethics, or publication ethics. (Please upload your review as an attachment if it exceeds 20,000 characters)

Reviewer #1: This is an interesting paper from a study which examines whether using an mRNA HPV test following a cytology negative result in women <40 years screened as part of the Norwegian cervical cancer screening programme could increase screening sensitivity, identifying more women who are at risk of developing CIN3+.

However, not all Figures are needed or clearly presented and correctly labelled. The methods do not contain sufficient details about the screening programme and the follow-up of women in the study including the small group who receive a positive HPV result following normal cytology who have their sample re-examined. The strengths and limitations of the study are not discussed.

I hope that my queries and suggestions are helpful.

Title
I would suggest rewording the title. It doesn’t make sense grammatically or describe the study very clearly.

Introduction
Line 44-46. Consider changing to ‘HPV testing is performed for the early detection of cervical disease, both as a reflex test following cytology or as a primary screening method.’
Line 47. Specificity for what? i.e. for identifying women with high-grade lesions, CIN, cancer or something else?
Line 52. You say ‘by 2022’ but it is already mid-way through. Do you mean the end of 2022? Was it previously cytology based (I assume?)
Line 55. I think you mean that it’s not just that the screening programme hasn’t transitioned to primary HPV testing for this group but that cytology will remain the primary screening tool for this younger age group?
Line 58. It is normal practice to avoid starting a sentence with a number.
Line 59. I believe that terminology is moving away from using ‘smear’.
Line 62-64. Consider rephrasing this last sentence as it is crucial here that the reader understands your main point: i.e., ‘younger women are screened using cytology – given the high number of precancerous lesions in this group an mRNA reflex test following a negative cytology result could be used as a quality assurance measure to increase screening sensitivity’.
Line 66: there should be no apostrophe in HPVs.
Line 70: you use the word ‘proven’. Suggest rewording to ‘shown’ or ‘found’
Line 71: consider using a different word than ‘tool’
Line 72: ‘Elevated risk of future’. In line 62 you infer they are ‘high risk’ but here you state low-risk population.
Line 73: Consider rewording this for clarity. Also, avoid the use of ‘we’ but state as ‘the aim of this study was…’.

Methods:
I understand from the methods section that your baseline samples are taken as part of routine screening in 2014-2017. However, you do not state how long the interval is between routine screens for women of this age or what happens to this interval if they have a positive cytology result.
Line 108: Your main outcome measure is CIN3+. But I do not understand from the Methods how this outcome is measured, when and in whom? Everyone presumably? But as part of their subsequent screen – and if that’s the case, surely not everyone has a subsequent screen? Some must be lost to follow up? In the Results you mention ‘follow-up through 2021’ but you need to explain more in the Methods what the follow-up means.
Line 106: I think you are saying that in women with a positive mRNA HPV result their index cytology (originally deemed as ‘normal’) is re-examined by a technician. But you do not then present the numbers of how many are deemed as ‘normal’ when re-examined? What then happens to those with an ‘abnormal’ result on re-examination in terms of their screening internal? Colposcopy? What is their CIN3+ outcome? And what happens or would happen to those who are HPV+ve but cytology ‘normal’ on second reading, is the interval to their next screen shorter?

Results
Figure 1: You split by the group that have mRNA testing and the group that don’t. Have you considered splitting first by cytology normal and cytology abnormal, then splitting the cytology normal into those tested for HPV and those not – this makes a more intuitive tree – but ends up with the same four groups represented at the bottom? The diagram needs and title and footnotes to explain abbreviations.
Line 136: Correction ‘a short time’
Line 138: Correction ‘nest’ to ‘next’
Figure 2: I have no idea what this figure means. It doesn’t add anything to the results so consider removing.
Figure 3: Without knowing anything about the follow-up of these women, the screening cycles etc. this seems hard to interpret. The labeling seems insufficient, for example these four groups are actually 1) normal cytology + mRNA HPV positive 2) abnormal cytology (no HPV testing) 3) Normal cytology (no HPV testing) 4) normal cytology + mRNA HPV negative.
There is no info in the methods about how these data were obtained or what the graph represents so consider removing this or labeling better.
Table 1: See my comment about labeling of Figure 3. You do not mention this table in the Results. I do not think that the label PPV is correct. PPV in a clinical setting means what is the probability, given your positive result, that you really are positive. The cytology ‘abnormal’ result would be considered a ‘positive’ result as would the mRNA HPV positive group, but the other groups would be considered as a ‘negative’ result. I think what these values really are is the percentage who go on to develop CIN3+ within 7 years of the baseline result (i.e. 2014-2021).

Discussion
Line 173: why is this in quotations?
Line 177: consider using ‘intense’ rather than aggressive?

You do not discuss the strengths or limitations of the study. For example, the fact that there is no statistical analysis.
Using mRNA HPV testing as a reflex test is only one way to change the screening pathway for this age group, it might be appropriate to suggest alternatives (such as AI to improve cytology) or suggest future research e.g. a cost-effectiveness analysis.

I’m not clear on what you are suggesting happens to women who are found cytology normal mRNA HPV positive – colposcopy or a shorter screening interval?

Line 210: You say 2.6% of cases in our study needed to be rescreened – i.e. 2.6% of samples with normal cytology but positive mRNA HPV have their original sample re-evaluated by a technician. Using the word ‘rescreened’ suggests they provide a new sample, but I don’t think from the methods that this is the case.

Line 218: There is no excessive workload in terms of the number of samples that need to be re-examined but if HPV testing were used as a reflex test then every cytology normal sample would need a reflex HPV test and this would increase the workload in the labs presumably as well as the cost of screening.

Reviewer #2: General comments;
This study showed that three type HPV mRNA test increases the sensitivity in cervical cancer screening program without an excessive workload. This is interesting and important study to prevent cervical cancer incidence in young women. The authors also said that detection of three HPV types (HPV types 16, 18, and 45) in this assay might be best choice in the screening of women of 25-39 years of age, since some previous studies suggested that 90% of cervical cancer are positive with HPV16, 18 or 45 in this age group.
However, this reviewer does not convince with this hypothesis that this three-HPV mRNA test is the best choice for the above mentioned purpose, since this study showed that the sensitivity of CIN3+ in the intervention group was merely 50% (14/28), although the sensitivity was high enough as it was 97.4% (1847/1896). The authors are likely to say no cancer were found in these CIN3+ cases in the intervention group. However, to prove this assay is the best choice, the authors should show most of 13 cervical cancer cases in the control group in this study are positive by this assay. If possible, they also should examine what HPV types are positive in 14 CIN3+ cases showing negative with this HPV test in the intervention group. Such data may be important to support the findings.

6. PLOS authors have the option to publish the peer review history of their article ([what does this mean?](https://journals.plos.org/plosone/s/editorial-and-peer-review-process#loc-peer-review-history)). If published, this will include your full peer review and any attached files.


If you choose “no”, your identity will remain anonymous but your review may still be made public.


**Do you want your identity to be public for this peer review?** For information about this choice, including consent withdrawal, please see our [Privacy Policy](https://www.plos.org/privacy-policy).

Reviewer #1: No

Reviewer #2: **Yes:** Toshiyuki Sasagawa

[NOTE: If reviewer comments were submitted as an attachment file, they will be attached to this email and accessible via the submission site. Please log into your account, locate the manuscript record, and check for the action link "View Attachments". If this link does not appear, there are no attachment files.]

While revising your submission, please upload your figure files to the Preflight Analysis and Conversion Engine (PACE) digital diagnostic tool, <https://pacev2.apexcovantage.com/>. PACE helps ensure that figures meet PLOS requirements. To use PACE, you must first register as a user. Registration is free. Then, login and navigate to the UPLOAD tab, where you will find detailed instructions on how to use the tool. If you encounter any issues or have any questions when using PACE, please email PLOS at [figures@plos.org](mailto:figures@plos.org). Please note that Supporting Information files do not need this step.

*In compliance with data protection regulations, you may request that we remove your personal registration details at any time.* [*(Remove my information/details)*](https://www.editorialmanager.com/pone/login.asp?a=r)*. Please contact the publication office if you have any questions.*
